# Comparative Analysis of Oral Microbiome in Indian Type 2 Diabetes Mellitus (T2DM) and Periodontitis Cohorts

**DOI:** 10.3390/diseases14020038

**Published:** 2026-01-23

**Authors:** Meenakshi Murmu, Rajshri Singh, Rajesh Gaikwad, Akshaya Banodkar, Sagar Barage, Preethi Sudhakara, Aruni Wilson Santhosh Kumar

**Affiliations:** 1 Amity Institute of Biotechnology, Amity University Maharashtra, Mumbai-Pune Expressway, Bhatan, Post-Somathne, Panvel 410206, India; meenakshi.murmu@s.amity.edu (M.M.); rsingh1@mum.amity.edu (R.S.); shbarage@mum.amity.edu (S.B.); 2 Department of Periodontology, Government Dental College and Hospital, Fort, Mumbai 400001, India; rajeshp.gaikwad@gov.in (R.G.); akshaya.bandodkar@gov.in (A.B.); 3 College of Medicine, University of Florida, Gainesville, FL 32610, USA; preethisudhakara@ufl.edu; 4 School of Medicine, California University of Science and Medicine, Colton, CA 92324, USA

**Keywords:** oral microbiome, gingival plaque, type 2 diabetes mellitus, periodontitis, 16S rRNA metagenomic sequencing

## Abstract

Background: Type 2 diabetes mellitus (T2DM) and periodontitis are highly prevalent immune-inflammatory diseases that interact bidirectionally. However, how early-onset T2DM, periodontitis, and adverse lifestyle behaviors collectively remodel the gingival plaque microbiome at the ecological network level remains poorly understood in Indian populations. Methods: A cross-sectional 16S rRNA gene (V3–V4) sequencing study was conducted on supragingival and subgingival plaque from 60 adults (30–40 years) recruited in Mumbai. Participants were categorized as healthy (H, *n* = 10), periodontitis (P, *n* = 10), T2DM (*n* = 20), and T2DM with periodontitis (T2DM_P, *n* = 20). Comprehensive demographic, anthropometric, metabolic, periodontal, dietary, lifestyle, and oral hygiene data were collected. Sequence data were processed using QIIME2–DADA2, followed by diversity, differential abundance, and genus-level co-occurrence network analyses (Spearman |r| ≥ 0.6, FDR < 0.05; core prevalence ≥ 70%). Results: α-diversity showed no marked depletion across groups, whereas Bray–Curtis β-diversity revealed significant global separation, with maximal dissimilarity between H and T2DM_P. Healthy individuals with favorable lifestyle behaviors harbored scaffold-forming taxa such as *Corynebacterium matruchotii*, *Lautropia mirabilis*, and *Capnocytophaga* spp. In contrast, P and T2DM_P groups showed enrichment of proteolytic, inflammation-adapted genera including *Porphyromonas*, *Tannerella*, *Treponema*, *Fretibacterium*, *Peptostreptococcus*, and *Selenomonas*. Network analysis revealed a shift from commensal-rich modular networks to densely connected, keystone-centered disease modules. Conclusion: Early-onset T2DM and periodontitis, particularly under adverse lifestyle behaviors, reorganize plaque microbial composition and interaction architecture rather than depleting diversity, highlighting plaque-based keystone taxa and networks as targets for microbiome-informed risk stratification and integrated medical–dental–lifestyle interventions.

## 1. Introduction

Type 2 diabetes mellitus (T2DM) and periodontitis are chronic immune-inflammatory conditions that frequently coexist and exacerbate each other through shared metabolic and inflammatory pathways [1]. Despite this well-recognized bidirectional relationship, oral health is rarely integrated into routine diabetes care, particularly in resource-constrained or underserved populations. According to the International Diabetes Federation (IDF) Atlas 11th edition 2025, an estimated 589 million adults (20–79 years) live with diabetes, with projections reaching approximately 853 million by 2050 [2]. India contributes disproportionately to this burden, with approximately 89.8 million adults affected in 2024 and a projected increase to approximately 156.7 million by 2050 [2], underscoring the urgent need to address diabetes complications beyond classical micro- and macrovascular complications [3]. T2DM is strongly linked to modifiable lifestyle factors, including excess adiposity, physical inactivity, unhealthy diet, smoking, and alcohol use, alongside non-modifiable risks such as family history, ethnicity, and gestational or polycystic ovarian syndromes [4,5].

Periodontitis, an advanced form of periodontal disease characterized by gingival inflammation, clinical attachment loss, and alveolar bone resorption, is increasingly recognized as the “sixth complication” of diabetes [1]. Metabolic disturbances associated with T2DM—particularly chronic hyperglycemia and insulin resistance—disrupt periodontal immune homeostasis and shift the host response toward a persistent hyper-inflammatory state [1,6]. Sustained hyperglycemia promotes the formation of advanced glycation end-products (AGEs), which activate receptors for AGE (RAGE)-mediated signaling pathways, resulting in NF-κB activation and exaggerated production of pro-inflammatory cytokines such as IL-1β, IL-6, and TNF-α [1]. Concurrent diabetes-associated immune dysfunction, including impaired neutrophil chemotaxis and phagocytosis, altered macrophage polarization, increased oxidative stress, and defective resolution of inflammation, leads to inefficient bacterial clearance coupled with enhanced collateral periodontal tissue damage [1]. These processes also promote osteoclastogenesis via an increased RANKL/OPG ratio, accelerating alveolar bone loss and impairing periodontal wound healing [1].

Chronic periodontal inflammation is further mediated by a complex milieu of cytokines and tissue-degrading enzymes (e.g., IL-1β, IL-6, TNF-α, IL-17, IL-23, prostaglandin E2, and matrix metalloproteinases, MMPs), which can disseminate into the systemic circulation, aggravate insulin resistance, and worsen glycemic control [7,8]. The IL-23/IL-17 (Th17) axis plays a central role in chronic periodontitis, wherein IL-23 sustains pathogenic Th17 responses and IL-17 amplifies neutrophil-driven inflammation and osteoclastogenesis, thereby promoting connective tissue breakdown and alveolar bone resorption [9]. Elevated circulating IL-6, C-reactive protein (CRP), and TNF-α levels have been reported in T2DM, further supporting a systemic inflammatory link between periodontal breakdown and metabolic dysregulation [7,10].

Epidemiological studies have shown that individuals with diabetes have approximately threefold higher risk of developing periodontitis than non-diabetics, while patients with periodontitis exhibit increased odds of incident T2DM and poorer glycemic control [11]. This reciprocal amplification is thought to be driven by a combination of hyperglycemia-induced host susceptibility (advanced glycation end products, oxidative stress, and altered neutrophil function) and dysbiotic periodontal microbiota that sustain chronic inflammation [12]. Improving periodontal health can yield modest but clinically relevant reductions in HbA1c, highlighting the potential of periodontal care as an adjunctive strategy for diabetes management [13].

The human oral cavity hosts one of the most diverse microbial ecosystems within the body, encompassing over 700 bacterial species distributed across various ecological niches, including saliva, tongue, buccal mucosa, and both supragingival and subgingival plaque [14]. In a healthy state, these microbial communities exist in a dynamic equilibrium shaped by host–microbe co-evolution, ecological succession, metabolic cross-feeding, and interspecies signaling. However, poor oral hygiene, carbohydrate-rich diets, systemic diseases such as T2DM, immune dysregulation, and lifestyle exposures such as tobacco use and alcohol consumption can disrupt this balance. Tobacco use and alcohol consumption further exacerbate periodontal dysbiosis in T2DM by reshaping the subgingival microbiota toward anaerobic, proteolytic, and pathogenic taxa, impairing host immune responses, and creating an inflammation-permissive niche that accelerates connective tissue breakdown and alveolar bone loss [15,16].

Among oral niches, gingival plaque biofilm is particularly informative due to its spatial stability, intimate contact with periodontal tissues, and ease of minimally invasive sampling [7]. Plaque therefore offers higher taxonomic and ecological resolution than saliva for linking local periodontal status with systemic metabolic risk [7]. Over the past decade, 16S rRNA amplicon sequencing studies in T2DM have predominantly focused on salivary microbiota in cohorts from the USA [17], Spain [18], China [19], Japan [20], and North India [21], reporting shifts toward saccharolytic and acidogenic genera with altered diversity and potential biomarkers [22,23]. Nevertheless, key gaps remain: (i) plaque-based microbial community profiles in adults with early-onset T2DM are under-characterized; (ii) few studies have simultaneously compare metabolically healthy and diabetic individuals with and without clinically defined periodontitis; and (iii) ecological interaction networks and keystone taxa associated with disease remain insufficiently explored, particularly in Indian populations, where dietary, cultural, and lifestyle factors may uniquely shape the oral microbiome.

To address these gaps, the research was conducted as a cross-sectional, comparative 16S rRNA gene sequencing study of supragingival and subgingival plaque from four well-defined adult groups such as periodontally and metabolically healthy individuals (H), periodontitis patients (P), T2DM patients (T2DM), and T2DM with periodontitis (T2DM_P). All participants were aged 30–40 years, an age window selected considering the increasing prevalence of early-onset T2DM in urban India. Comprehensive demographic, anthropometric, clinical, and lifestyle data including BMI, tobacco and alcohol exposure, dietary patterns, physical activity, and diabetes duration were systematically recorded to contextualize microbiome variations within a detailed cardiometabolic and behavioral framework.

Using high-throughput next-generation sequencing of the V3-V4 region of the 16S rRNA gene combined with robust bioinformatics and statistical approaches (QIIME2–DADA2, rarefaction, α/β-diversity, PERMANOVA, CLR-based differential abundance, and correlation-based co-occurrence networks), this study aimed to (i) characterize plaque microbiota at the phylum, genus, and species levels; (ii) compare intra- and inter-group diversity across H, P, T2DM, and T2DM_P; and (iii) identify keystone taxa and interaction modules associated with metabolic dysregulation, periodontal destruction, or their comorbidity. By integrating clinical, lifestyle, and microbial data, this study sought to elucidate how metabolic and periodontal risk factors collectively shape plaque ecosystems and identify microbial signatures and network hubs that may inform risk stratification and targeted prevention strategies in populations at high risk of diabetes–periodontitis comorbidity.

## 2. Methods

### 2.1. Study Design and Sample Size

This exploratory, cross-sectional, comparative pilot study was designed to characterize the oral microbiome of Indian adults with early-onset Type 2 Diabetes Mellitus (T2DM) and its comorbid condition, periodontitis, in comparison with healthy subjects. Participants were recruited from the Government Dental College and Hospital (GDCH), Mumbai, India.

As this study represents an initial, exploratory investigation of oral microbiome differences among early-onset adults with T2DM, T2DM with periodontitis (T2DM_P), periodontitis (P), and healthy (H) individuals, an a priori power calculation was not performed. Instead, a balanced, stratified sampling strategy was implemented to ensure phenotypic representativeness across study groups while preserving statistical validity for non-parametric and multivariate microbiome analyses [24]. Consistent with prior oral microbiome research, small but well-characterized phenotype cohorts have been shown to capture robust community-level shifts and large ecological effect sizes when analyzed using distance-based, permutation-driven frameworks. Accordingly, a total of 60 participants were included, and PERMANOVA power and sensitivity were evaluated using a distance-matrix simulation framework specifically developed for marker-gene microbiome studies based on pairwise dissimilarities [25]. This approach is recommended because PERMANOVA power is determined by realistic within-group dispersion and between-group separation, rather than sample size alone [26]. Furthermore, comparable oral microbiome studies in T2DM populations have successfully detected compositional differences using cohort designs of similar scale, supporting the adequacy of the present study design [27]. Epidemiological data from an urban Mumbai cohort reported that the probability of T2DM in adults (male/female) aged 30–34 and 35–39 years was 0.18 (95% CI: 0.08, 0.38) and 0.36 (95% CI: 0.25, 0.48), respectively [28]. This rising prevalence of early-onset T2DM justifying our focus on this age window to capture microbiome perturbations associated with metabolic dysregulation. The study protocol was obtained ethical approval from the Institutional Ethics Committee, GDCH, Mumbai (Reference No. IEC2597/2025). Written informed consent was obtained from all participants prior to clinical data recording and biospecimen collection, in accordance with ethical and confidentiality standards.

### 2.2. Subject Recruitment and Inclusion/Exclusion Criteria

All participants were 30–40 years of age range. To minimize confounding factors, the participants were included if they met the following criteria [29,30]. For each participant, all periodontal examination was performed by periodontitis at the Department of Periodontology, GDCH, Mumbai, including assessments of probing pocket depth (PD), bleeding on probing (BOP), clinical attachment loss (CAL). A calibration meeting was held prior to study initiation and follow-up meetings during the study. Subjects were periodontally classified according to the 2017 Workshop on Periodontal Diseases and Conditions [31]. For comparative analyses between health and disease, only participants with clear periodontal status, either periodontally healthy or periodontitis were selected.

A total of 60 subjects were recruited and categorized into four groups: healthy individuals (H; *n* = 10), patients diagnosed with periodontitis (P; *n* = 10), T2DM patients with HbA1c > 7% (T2DM, *n* = 20); and T2DM patients with periodontitis (T2DM_P, *n* = 20). The exclusion criteria comprised (i) presence of systemic diseases and autoimmune disorders; (ii) use of anti-inflammatory drugs, antibiotics, or oral probiotics, within six months prior to enrollment; and (iii) pregnant or lactating mother.

All participants completed a structured questionnaire via face-to-face interview. Demographic, anthropometric, clinical, and lifestyle information, including body-mass index (BMI), smoking, tobacco and alcohol consumption status, dietary patterns, diabetes duration, medication, family history of diabetes, were documented using a standardized pro-forma (Supplementary Table S1), in accordance with established epidemiological protocols [32].

### 2.3. Sample Collection

Two site-specific oral biofilm samples supragingival and subgingival plaque were collected from each participant to represent both health-associated and disease-associated microbial niches. Participants were instructed to fast for at least 3 h and abstain from oral hygiene procedures, smoking and chewing prior to sampling to minimize transient microbiota fluctuations [29].

For the healthy (H) and T2DM groups, supragingival plaque was collected from the buccal surfaces of molars and premolars [22,30]. For periodontitis (P) and T2DM_P groups, subgingival plaque was collected from two teeth with the deepest pocket probing depths (≥5 mm), following established clinical protocols [29,33].

Plaque was carefully scrapped using sterile Nichrome Loop-D-3, LA051 (HiMedia Laboratories Pvt. Ltd., Thane, India) and immediately suspended in 1 mL sterile phosphate-buffered saline (PBS; pH 7.4) in pre-labeled 2 mL microtubes. The tubes were sealed with parafilm and coded using anonymized subject identifiers. Samples contaminated with blood were excluded. All samples were stored at −80 °C until DNA extraction. Blank swab and PBS negative controls were included throughout to monitor sterility and prevent cross-contamination [29].

### 2.4. Microbial DNA Isolation, Amplification and Sequencing

Genomic DNA was extracted using the QIAamp PowerFecal Pro DNA Kit (QIAGEN GmbH, Cat No. 51804, Hilden, Germany) with bead-beating, according to the manufacturer’s instructions. DNA concentration and purity were assessed using NanoDrop One (Thermo Fisher Scientific, Waltham, MA, USA) and Qubit dsDNA HS Assay (Thermo Fisher Scientific, Waltham, MA, USA). An aliquot of the extracted DNA was electrophoresed along with a 1 kb ladder on a 0.8% agarose 1X TAE gel and stained with SYBR safe to visualize the nucleic acid bands. Purified DNA was stored at −20 °C until amplification.

The V3-V4 hypervariable region of the bacterial 16S rRNA gene was amplified using Illumina overhang adapters appended to gene-specific primers described by Klindworth et al. [34]. The full-length primer sequences, using standard IUPAC nucleotide nomenclature (N = A/T/G/C, W = A/T, H = A/C/T, V = A/C/G), targeting the V3–V4 region were:

Forward: 5′TCGTCGGCAGCGTCAGATGTGTATAAGAGACAGCCTACGGGNGGCWGCAG3′.

Reverse: 5′GTCTCGTGGGCTCGGAGATGTGTATAAGAGACAGGACTACHVGGGTATCTAATCC3′.

PCR was performed in 25 µL reactions containing 2.5 µL of template DNA (12.5 ng/µL), 5 µL each of 1 µM forward and reverse primers, and 12.5 µL of 2× KAPA HiFi HotStart ReadyMix [34]. Amplicon product was validated on 0.8% agarose gel, co-electrophoresis with 1 kb ladder and purified using AMPure XP beads kit according to the manufacturer’s instructions. Indexed libraries were prepared using the Nextera XT Index Kit (Illumina: Index 1 primers (N7XX: FC-131-1001; and Index 2 Primers (S5XX: FC-131-1001). Library quality and fragment size distribution (∼630 bp) were assessed using an Agilent Bioanalyzer TapeStation (D1000/HSD1000 ScreenTape, Agilent Technologies Inc., Böblingen, Germany). Equimolar pooled libraries were sequenced on the Illumina MiSeq platform (2 × 300 bp, MiSeq Reagent kit v2, 500 cycle) at miBiome Therapeutics LLP, Mumbai, India.

### 2.5. Bioinformatics Processing

Raw paired-end FASTQ files were processed using Quantitative Insights into Microbial Ecology pipeline (QIIME2) pipeline (https://qiime2.org/, accessed on 10 May 2025) with the DADA2 plugin for quality filtering (Q = 20), denoising, chimera removal, and amplicon sequence variant (ASV) inference [35]. Taxonomic assignment was performed using Assign-Taxonomy-with-BLAST script to query the HOMD_16S_rRNA_RefSeq_V15.23 database (https://www.homd.org/, accessed on 10 May 2025). Low-abundance taxa (present in <10% of samples) were filtered, yielding 828 taxa, sequencing depth of 40,000 reads per sample was standardized by rarefaction, and processed data were imported into R using the phlyoseq. package. Visualization was performed using ggplot2 (R v3.5.1) [36].

### 2.6. Statistical Analysis

Statistical analyses were conducted using R software (R v3.5.1; https://www.r-project.org/, accessed on 20 May 2025). Descriptive statistics (mean, standard deviation, minimum, median, and maximum) were calculated for each group and variable. PPD and CAL were expressed in millimeters, while BOP, PI, and relative abundance were expressed as percentages. Alpha diversity was compared using the Kruskal–Wallis test, beta diversity using Bray–Curtis dissimilarity with PERMANOVA, and microbiome-clinical associations were evaluated using canonical correspondence analysis (CCA). Differential abundance analysis employed centered log-ratio (CLR) transformation, and genus-level co-occurrence networks were constructed using Spearman’s correlation (*p* < 0.05, |r| ≥ 0.6), with Benjamini–Hochberg FDR correction applied. Statistical significance was set at *p* < 0.05 [37].

## 3. Results

### 3.1. Demographic and Clinical Characteristics

Across the 60 participants, the four study groups were well matched for age and sex. Mean (±SD) ages were 36.7 ± 3.3 years for H (*n* = 10), 34.9 ± 3.2 years for P (*n* = 10), 37.0 ± 2.8 years for T2DM (*n* = 20) and 36.8 ± 2.6 years for T2DM_P (*n* = 20), with no apparent between-group differences. The proportion of males was comparable (H 50%, P 60%, T2DM 55%, T2DM_P 50%). Body weight and BMI differed markedly. Mean (±SD) weights were 66.5 ± 14.6 kg (H), 64.1 ± 8.3 kg (P), 76.8 ± 10.6 kg (T2DM) and 75.4 ± 9.9 kg (T2DM_P). BMI categories showed strong group separation (χ^2^, *p* < 0.001): excess adiposity (overweight and obese) was present in (18/20; 90%) T2DM subjects and (13/20; 65%) T2DM_P subjects, but in (5/10; 50%) P subjects and in none of the H subjects, who were all normal weight (Supplementary Table S1).

The healthy (H) group represented a metabolically low-risk reference, characterized by normal BMI, absence of tobacco or alcohol exposure, higher brushing frequency, regular moderate-to-vigorous physical activity, and favorable dietary patterns. No systemic comorbidities or oral symptoms were documented (Supplementary Table S1).

In the periodontitis (P) group exhibited periodontal disease largely independent of metabolic dysfunction, with normal-to-overweight BMI distribution but significantly poorer oral hygiene, high prevalence of tobacco/alcohol exposure (90%), habitual sugar-sweetened beverage intake, and universal gingival inflammation, halitosis, and frequent deep pockets or tooth loss (Supplementary Table S1).

The T2DM group showed a marked metabolic burden, with 90% overweight/obese status, elevated HbA1c (mean ≈ 6.5%), frequent cardiometabolic and neuropathic symptoms, and higher tobacco/alcohol exposure, but minimal periodontal pathology, constituting a metabolically compromised yet periodontally healthy diabetic reference group (Supplementary Table S1).

The T2DM_P group demonstrated the most severe combined phenotype, characterized by high obesity prevalence (65%), extensive tobacco/alcohol exposure (75%), frequent systemic comorbidities, and significantly greater periodontal disease severity, including deep pockets, calculus accumulation, halitosis, missing teeth, and xerostomia (Supplementary Table S1).

### 3.2. Periodontal Assessment

A quantitative comparison of periodontal clinical parameters (Table 1) revealed significant intergroup differences across all indices (*p* < 0.0001) [33]. The probing depth (PD), clinical attachment loss (CAL), periodontal pocket depth (PPD), bleeding on probing (BOP), and plaque index (PI) were substantially elevated in the periodontitis (P) and T2DM_P groups compared to the healthy (H) and T2DM groups [8]. This indicates a more advanced periodontal deterioration and inflammatory burden in these cohorts. Post hoc pairwise analyses corroborated this gradient of disease severity, with the pronounced structural damage and inflammation consistently observed in P and T2DM_P, and relatively limited periodontal involvement in H and T2DM.

### 3.3. Summary of Sequencing Data

**Figure 1:**
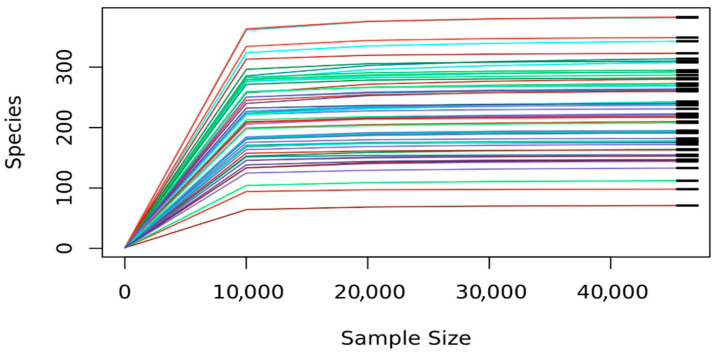
Rarefaction curves showing sequencing depth across study groups. The curves plateau at 40,000 reads per sample, indicating sufficient sequencing coverage for reliable diversity estimation.

A total of 10,144,832 raw paired-end reads were generated from 60 plaque samples (mean ± SD = 169,080.5 ± 42,550.4 reads/sample; range 70,096–238,187). Following rigorous quality filtering, chimera removal, and read merging, 6,148,666 high-quality reads were retained (mean = 102,477.8 ± 31,122.1 reads per sample). The DADA2 denoising process produced amplicon sequence variants (ASVs), which were taxonomically classified using the Human Oral Microbiome Database (HOMD v15.23), resulting in the identification of 4862 taxa across 12 phyla, 25 classes, 36 orders, 52 families, 112 genera, and 295 species. No significant variation in sequencing depth was observed across the study groups, ensuring balanced library sizes and minimizing bias in diversity estimation. Rarefaction curves reached a plateau across all samples (Figure 1), indicating sufficient sequencing depth and coverage for a reliable α-diversity assessment. All subsequent analyses were conducted on rarefied data (40,000 reads per sample) to ensure a uniform sampling depth across groups. For community composition (PCoA) and differential abundance analyses, data were normalized using centered log-ratio (CLR) transformation to account for the compositional nature of microbiome data, as implemented in the phyloseq package in R [36,37,38]. The processed dataset exhibited high coverage, evenness, and taxonomic resolution, providing a robust foundation for downstream ecological and comparative analyses of the oral microbiota. All raw sequence data have been deposited in the NCBI Sequence Read Archive (SRA) under BioProject accession number PRJNA1240053 (https://www.ncbi.nlm.nih.gov/sra, accessed on 30 April 2025).

To comprehensively evaluate variations in the oral microbial communities among study groups, α-diversity analysis, principal coordinate analysis (PCoA) based on Bray–Curtis distances, canonical correspondence analysis (CCA), stacked taxonomic profiling, and co-occurrence network analysis were performed to identify potential keystone genera.

### 3.4. Diversity Analysis

**Figure 2:**
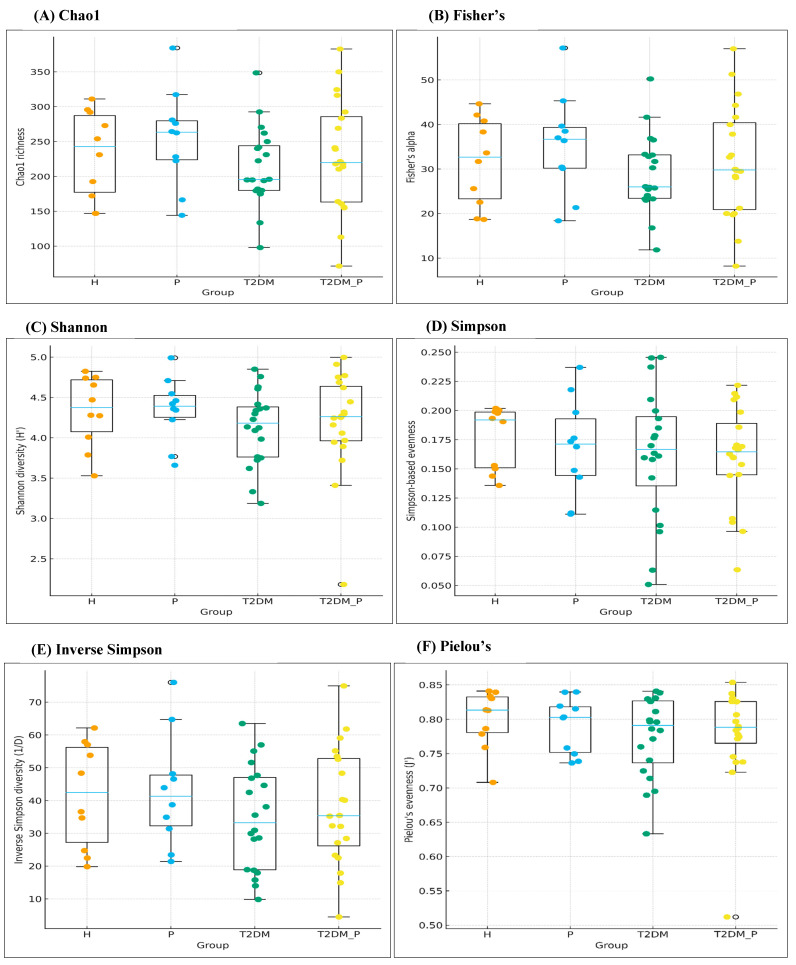
α-diversity of gingival plaque microbiota across clinical groups.: (**A**) Chao1 richness, (**B**) Fisher’s α, (**C**) Shannon diversity (H′), (**D**) Simpson-based evenness, (**E**) Inverse Simpson diversity (1/D) and (**F**) Pielou’s evenness (J′) for Healthy (H), Periodontitis (P), T2DM and T2DM_P subjects. Boxplots depict medians and interquartile ranges with whiskers extending to 1.5× IQR; individual samples are overlaid as jittered points. All six indices show broadly overlapping distributions and non-significant Kruskal–Wallis tests (*p* > 0.05 for all comparisons), indicating preserved within-sample richness, diversity and evenness across clinical groups.

Six α-diversity indices were employed to assess within-group microbial diversity, encompassing richness (Chao1, Fisher’s α), diversity (Shannon H′, Inverse Simpson 1/D) and evenness (Simpson-based evenness, Pielou’s J′) (Figure 2A–F; Supplementary Table S2). Regarding the richness, the Chao1 medians [IQR] were 242.6 [177.3–287.3] in H, 263.4 [223.9–279.8] in P, 195.5 [179.9–244.0] in T2DM and 219.8 [163.3–285.7] in T2DM_P (Figure 2A). Fisher’s α exhibited a similar pattern, with values of 32.7 [23.3–40.2], 36.7 [30.2–39.3], 26.0 [23.4–33.2] and 29.8 [20.9–40.4] in H, P, T2DM and T2DM_P, respectively (Figure 2B). Although the periodontitis groups demonstrated slightly higher central richness estimates compared to the healthy and T2DM groups, the interquartile ranges overlapped considerably, and non-parametric Kruskal–Wallis tests for Chao1 and Fisher’s α were not significant (H = 2.21, *p* = 0.53; H = 2.31, *p* = 0.51). In terms of diversity, Shannon H′ values were closely aligned across groups (H: 4.38 [4.07–4.72], P: 4.39 [4.25–4.53], T2DM: 4.18 [3.76–4.38], T2DM_P: 4.26 [3.96–4.64]; Figure 2C), with no discernible separation between distributions (Kruskal–Wallis H = 2.29, *p* = 0.51). Inverse Simpson 1/D values indicated slightly lower medians in T2DM (33.2 [18.9–47.0]) and T2DM_P (35.3 [26.2–52.8]) relative to H (42.5 [27.2–56.2]) and P (41.3 [32.3–47.8]), yet this remained within the healthy range, and the overall test did not reach significance (H = 2.09, *p* = 0.56; Figure 2E). Evenness was also conserved. Simpson-based evenness medians [IQR] were 0.19 [0.15–0.20] for H, 0.17 [0.14–0.19] for P, 0.17 [0.14–0.19] for T2DM and 0.16 [0.14–0.19] for T2DM_P (Figure 2D; H = 0.59, *p* = 0.90). Pielou’s J′ values were similar across all groups (H: 0.81 [0.78–0.83], P: 0.80 [0.75–0.82], T2DM: 0.79 [0.74–0.83], T2DM_P: 0.79 [0.77–0.83]; Figure 2F; H = 1.65, *p* = 0.65), indicating the absence of dominance by a few taxa under any clinical condition.

**Figure 3:**
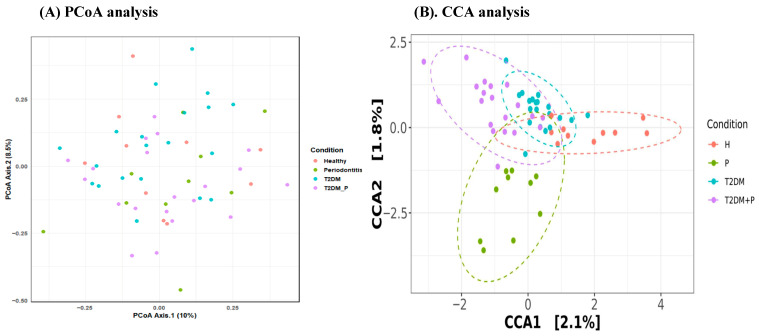
β-diversity of gingival plaque microbiota across clinical groups: (**A**) PCoA of Bray–Curtis dissimilarities showing ordination of plaque samples from Healthy (H, orange), Periodontitis (P, green), T2DM (blue) and T2DM_P (purple) participants. PCoA Axis 1 and Axis 2 explain 10.0% and 8.6% of the total variance, respectively. (**B**) CCA constrained by clinical condition, with the first two canonical axes (CCA1, 2.1%; CCA2, 1.8%) displayed. Ellipses represent the 95% confidence region for each group. PERMANOVA on Bray–Curtis distances and the global CCA test demonstrate significant differences in community composition among the four conditions (*p* < 0.05 after Benjamini–Hochberg correction, all padj ≥ 0.108), indicating that metabolic and periodontal status together shape the overall structure of the plaque microbiome.

The β-diversity structure of the plaque microbiome was initially evaluated through principal coordinates analysis (PCoA) based on Bray–Curtis dissimilarities (Figure 3A). The first two axes accounted for 10.0% and 8.6% of the total variance, respectively, and demonstrated a discernible, albeit incomplete, separation of samples according to clinical condition. Healthy (H) and periodontitis (P) samples occupied partially overlapping regions, whereas T2DM and T2DM_P samples shifted towards distinct areas of ordination space, consistent with the compositional restructuring of plaque communities under metabolic stress. A PERMANOVA on Bray–Curtis distances confirmed that community composition differed significantly among the four groups (PERMANOVA, *p* < 0.05 after Benjamini–Hochberg correction, all padj ≥ 0.108), indicating that clinical status accounts for a non-trivial fraction of the variation in plaque microbiota composition between samples.

To further investigate disease-associated gradients in community structure, we performed canonical correspondence analysis (CCA) with the clinical group as the constraining variable (Figure 3B). The two canonical axes explained 2.1% and 1.8% of the constrained variance, respectively, and enhanced the separation between conditions. Periodontitis (P) samples formed a distinct cluster along negative CCA2 scores, whereas T2DM samples were more tightly grouped in the upper central region, and T2DM_P samples extended along negative CCA1 with positive CCA2 scores. Healthy (H) samples were primarily distributed towards positive CCA1 values. This pattern suggests that periodontal inflammation and hyperglycemic status exert partially independent but interacting effects on plaque community composition. The overall CCA model was statistically significant (*p* = 0.001), supporting a robust association between clinical conditions and microbial community structure, despite the relatively modest proportion of variance captured by the two axes.

### 3.5. Overview of Microbiome Characterization at Phylum, Genus, and Species Level

**Figure 4:**
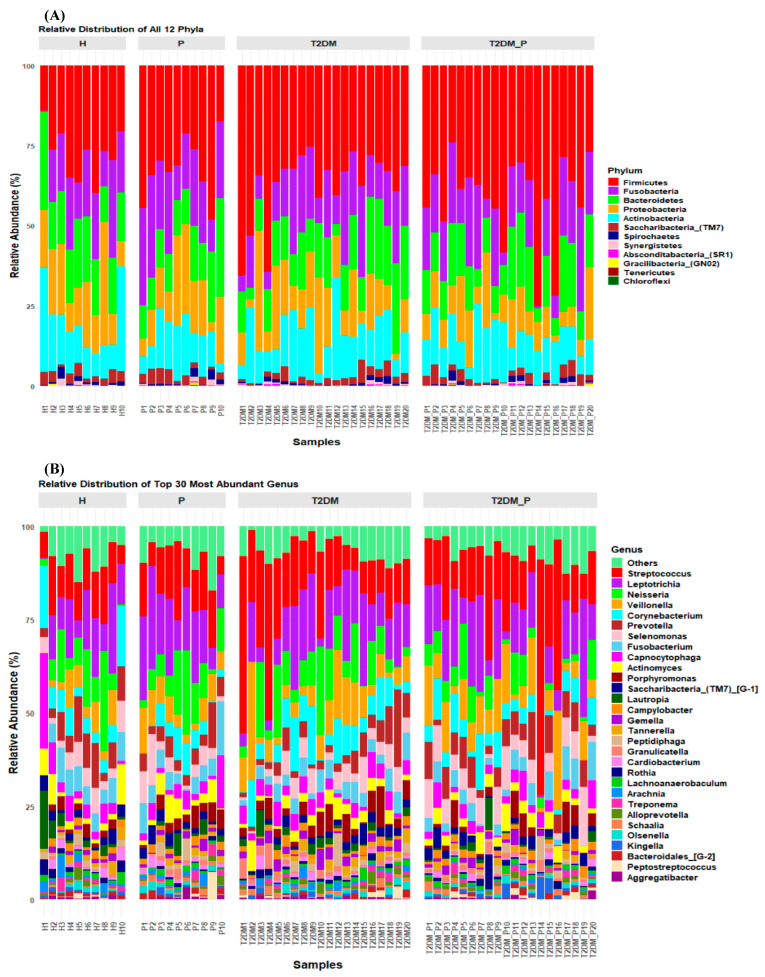
Taxonomic resolution and comparison at the phylum and genus level: (**A**) Phylum Relative abundance distribution across all groups. (**B**) Genus-level relative abundance distribution, with “Others” comprising low-abundance taxa.

At the phylum level, taxonomic profiling of supragingival and subgingival plaque communities (Figure 4A) identified six dominant lineages: *Firmicutes* (36.17%), *Fusobacteria* (17.88%), *Bacteroidetes* (15.26%), *Proteobacteria* (13.55%), *Actinobacteria* (13.26%), and *Saccharibacteria*_(TM7) (2.53%), collectively comprising nearly the entire bacterial community. Phyla of the lower abundance included *Spirochaetes* (0.80%), *Synergistetes* (0.32%), *Absconditabacteria*_(SR1) (0.10%), *Gracilibacteria*_(GN02) (0.06%), *Tenericutes* (0.04%), and *Chloroflexi* (0.03%). Comparative analysis revealed a significant enrichment of *Firmicutes* in both diabetic groups-T2DM (37.24%) and T2DM_P (41.38%), while *Fusobacteria* was most abundant in the periodontitis (P) (21.43%) and T2DM_P (17.85%) groups. Conversely, the healthy (H) group exhibited higher proportions of *Bacteroidetes* (18.22), *Proteobacteria* (16.93%), and *Actinobacteria* (15.79%). Among minor lineages (<1%), *Choloroflexi* was notably elevated in the periodontitis (P) and T2DM_P groups (Figure 4A).

At the genus level, the core microbiome across all groups was dominated by *Streptococcus* (15.87%), *Leptotrichia* (12.55%), *Neisseria* (9.11%), *Veillonella* (6.17%), *Corynebacterium* (5.37%), *Prevotella* (4.65%), *Selenomonas* (4.19%), *Fusobacteria* (3.82%), *Capnocytophaga* (3.79%), *Actinomyces* (3.29%), *Porphyromonas* (3.10%), *Lautropia* (1.54%), *Campylobacter* (1.42%), *Peptidiphaga* (1.35%), and *Gemella* (1.30%) (Figure 4B; Supplementary Table S3).

Differential mean relative abundance analysis (Table 2; Supplementary Table S4) demonstrated that the healthy (H) group was significantly enriched in *Corynebacterium* (6.94%), *Prevotella* (6.91%), *Capnocytophaga* (6.07%), *Fusobacterium* (5.58%), and *Actinomyces* (3.64%). The periodontitis (P) group showed increased abundances of *Leptotrichia* (15.99%), *Neisseria* (9.18%), *Porphyromonas* (3.57%), *Tannerella* (1.49%), and *Peptidiphaga* (1.36%). The T2DM group was enriched in *Porphyromonas* (3.57%), *Gemella* (1.85%), *Granulicatella* (1.55%), *Alloprevotella* (0.89%), and *Abiotrophia* (0.55%), whereas the T2DM_P group harbored higher levels of *Streptococcus* (17.96%), *Veillonella* (9.09%), *Selenomonas* (6.20%), *Campylobacter* (1.63%), and *Kingella* (1.14%). Notably, *Absconditabacteria*_(SR1)_[G-1], *Phocaeicola*, *Acidipropionibacterium*, and *Megasphaera* were consistently detected in both diabetic groups, whereas *Filifactor* was shared between the healthy (H) and periodontitis (P) groups. In contrast, *Scardovia*, *Bacteroides*, *Butyrivibrio*, *Bulleidia* and *Pyramidobacter* were absent from the healthy (H) group, while *Slackia* and *Shuttleworthia* were exclusively identified in healthy (H) plaques.

**Figure 5:**
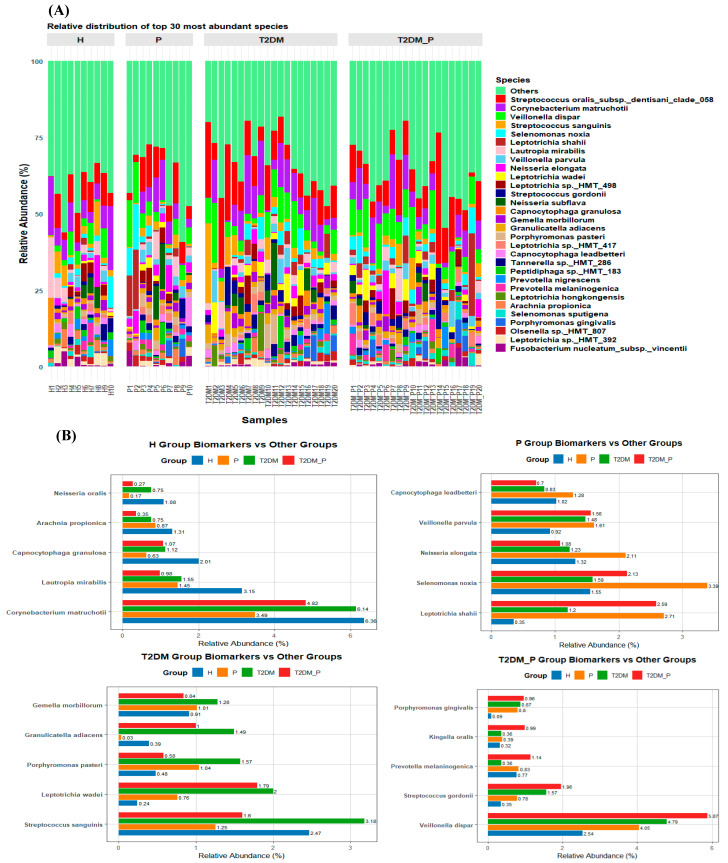
Species-level relative abundance and comparison of the five most abundant biomarker species across all groups: (**A**) Relative abundance distribution of the 30 most abundant species across all groups. (**B**) Comparative relative abundance of top five biomarker species, identified as: *Corynebacterium matruchotii* in healthy (H), *Selenomas noxia* in periodontitis (P), *Streptococcus sanguinis* in Type 2 Diabetes Mellitus (T2DM), *Veillonella dispar* in T2DM-associated periodontitis (T2DM_P).

Species-level profiling (Figure 5A) revealed that the predominant taxa across all study groups were *Streptococcus oralis_*subsp.*_dentisani_clade*_058 (6.57%), *Corynebacterium matruchotii* (9.09%), *Veillonella dispar* (7.98%), *Streptococcus sanguinis* (3.80%), *Selenomonas noxia* (3.23%), *Leptotrichia shahii* (3.04%), *Lautropia mirabilis* (2.79%), *Veillonella parvula (*2.53%), *Leptotrichia wadei* (2.46%), *Streptococcus gordonii* (2.35%), *Neisseria subflava* (1.29%), *Capnocytophaga granulosa* (1.21%), *Gemella morbillorum* (1.01%), *Granulicatella adiacens* (0.98%), *Porphyromonas pasteri* (0.94%), *Capnocytophaga leadbetteri* (0.92%), *Prevotella nigrescens* (0.79%), *Prevotella melaninogenica* (0.82%), *Leptotrichia hongkongensis* (0.77%), *Arachnia propionica* (0.75%), *Selenomonas sputigena* (0.71%), and *Porphyromonas gingivalis* (0.70%). Collectively, these taxa accounted for 55.43% of the total community abundance (Supplementary Table S5).

Differential abundance analysis (Table 3; Supplementary Table S6) revealed distinct species-level signatures among study groups (Figure 5B). In the Healthy (H) group, *Corynebacterium matruchotii* (6.36%), *Lautropia mirabilis* (3.15%), *Capnocytophaga granulosa* (2.01%), *Arachnia propionica* (1.31%), and *Neisseria oralis* (1.08%) were the most enriched taxa. The Periodontitis (P) group exhibited higher abundances of *Selenomonas noxia* (3.39%), *Leptotrichia shahii* (2.71%), *Neisseria elongata* (2.11%), *Veillonella parvula* (1.61%), and *Capnocytophaga leadbetteri* (1.21%). In contrast, the T2DM group was enriched in *Streptococcus sanguinis* (3.18%), *Leptotrichia wadei* (2.00%), *Porphyromonas pasteri* (1.57%), and *Granulicatella adiacens* (1.49%), and *Gemella morbillorum* (1.28%). The T2DM_P group was characterized by higher relative abundances of *Veillonella dispar* (5.87%), *Streptococcus gordonii* (1.96%), *Prevotella melaninogenica* (1.14%), *Kingella oralis* (0.99%), and *Porphyromonas gingivalis* (0.96%).

Notably, *Streptococcus oralis_*subsp.*_dentisani_*clade_058 was consistently detected in both diabetic groups, suggesting a potential shared microbial adaptation to the diabetic oral environment. In contrast, several species including *Scardovia wiggsiae*, *Capnocytophaga gingivalis*, *Bacteroides heparinolyticus*, *Actinomyces israelii*, and *Neisseria cinerea*, *Pyramidobacter piscolens*, *Bulleidia extructa*, and *Treponema pectinovorum* were completely absent from the Healthy (H) microbiome, underscoring potential disease-associated exclusively.

### 3.6. Co-Occurrence Network Analysis and Identification of Keystone Genus

**Figure 6:**
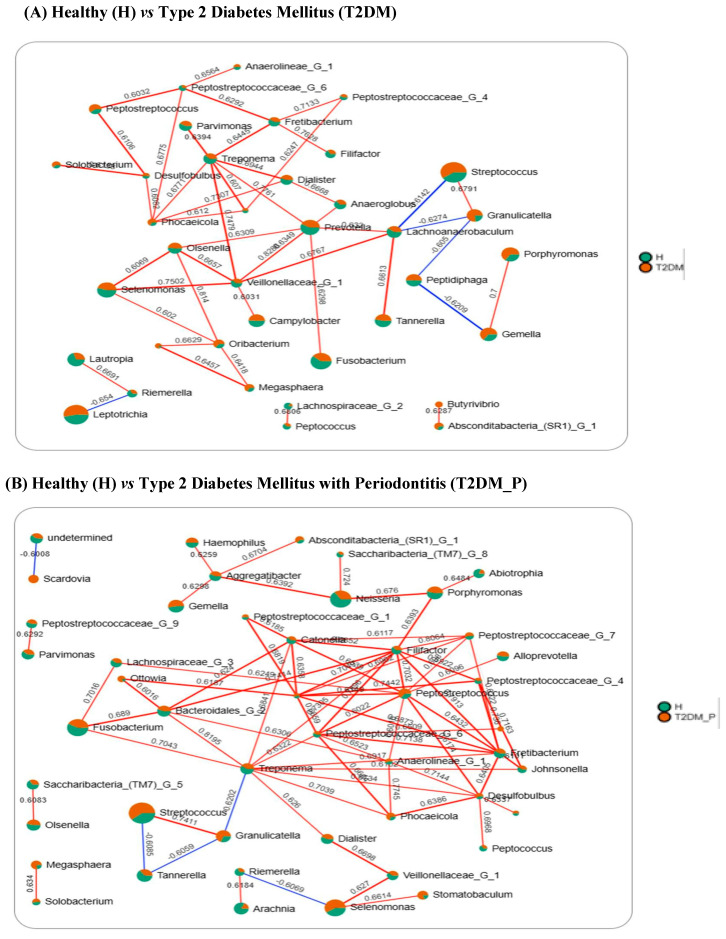
Genus-level co-occurrence networks of gingival plaque microbiota in healthy (H) versus metabolic–periodontal (T2DM, T2DM_P) disease: (**A**) Network comparing H, green segments) vs. T2DM (orange segments) groups. (**B**) Network comparing H (green) and T2DM_P (orange). Each node represents a bacterial genus; node pie charts indicate the relative contribution of each group to the total abundance of that genus. Edges connect genera represents statistically significant correlations (Spearman, |r| ≥ 0.6, *p* < 0.05) revealed group-specific topologies; red and blue edges represent positive and negative associations, respectively, and edge labels show correlation coefficients. The H-T2DM network shows a shared core community with a discrete disease-associated module of anaerobic genera, whereas the H-T2DM_P network is denser and dominated by strongly co-occurring periodontopathogens and bridging taxa, consistent with a more tightly integrated dysbiotic consortium in subjects with combined T2DM and periodontitis.

To examine ecological interrelationships and community organization, genus-level co-occurrence networks were constructed for H vs. T2DM and H vs. T2DM_P using Spearman’s rank correlations (|r| ≥ 0.6, *p* < 0.05) with Benjamini–Hochberg FDR correction (adjusted *p* < 0.05) (Figure 6A,B). Only genera present in ≥70% of samples within each group were retained to enhance network stability and minimize the stochastic effects of rare taxa. The final networks, therefore, consisted exclusively of robust, statistically supported associations; edge labels denoted correlation coefficients, and red and blue edges represented positive and negative correlations, respectively. Topological properties (degree and betweenness centrality, clustering coefficient) were then used to identify highly connected “keystone” genera acting as hubs or bridges within the microbial ecosystem, thereby capturing ecological importance beyond relative abundance [39,40].

#### 3.6.1. H vs. T2DM

In the T2DM network (Figure 6A), a densely interconnected positive correlation module was dominated by putative pathogens (orange), including *Streptococcus*, *Granulicatella*, *Porphyromonas*, *Gemella*, *Oribacterium*, *Phocaeicola*, *Megasphaera*, *Solobacterium*, and *Peptostreptococcus*. In contrast, Healthy (H) enriched taxa (shown in green color) such as *Treponema*, *Prevotella*, *Fusobacterium*, *Lautropia*, *Lachnoanaerobaculum*, *Filifactor*, *Riemerella*, *Dialister*, *Olsenella*, *Veillonellaceae*_G_1, *Lachnoanaerobaculum*, *Parvimonas*, *Tannerella*, *Fretibacterium*, *Anaeroglobus*, *Lachnospiraceae*_G_2, *Peptococcus*, *Peptostreptococcaceae_*G_4 and *Anaerolineae*_G_1 were clustered into a cooperative commensal hub.

Within this framework, *Treponema* exhibited the highest degree centrality and acted as a keystone connector, showing strong positive associations with *Prevotella* (r = 0.776), *Veillonellaceae_*G_1 (r = 0.747), *Dialister* (r = 0.694), *Phocaeicola* (r = 0.677), *Fretibacterium* (r = 0.644) and *Parvimonas* (r = 0.639). *Veillonellaceae_G*_1 further demonstrated robust co-occurrence with *Prevotella* (r = 0.828), *Selenomonas* (r = 0.750), *Olsenella* (r = 0.666), *Lachnoanaerobaculum* (r = 0.676), and *Camplyobacter* (r = 0.603). Additionally, *Fretibacterium* strongly co-occurred with *Filifactor* (r = 0.762), *Peptostreptococcaceae*_G_4 (r = 0.713), and *Peptostreptococcaceae*_G_6 (r = 0.629).

Antagonistic correlations, which reflect ecological competition or niche exclusion, were also evident and contributed to the differentiation of the keystone species. *Lachnoanaerobaculum* correlated positively with *Tannerella* (r = 0.661), *Prevotella* (r = 0.623), *Veillonellaceae*_G_1 (r = 0.676), but negatively with *Streptococcus* (r = –0.614) and *Granulicatella* (r = −0.627), indicating potential antagonism between commensal and opportunistic taxa. Similarly, *Gemella* was positively linked to *Porphyromonas* (r = 0.700) but negatively to *Peptidiphaga* (r = −0.621); *Riemerella* correlated positively with *Lautropia* (r = 0.669) and negatively with *Leptotrichia* (r = −0.654). Notably, the rare genus *Butyrivibrio*, which was exclusive to T2DM, exhibited a significant positive correlation with *Absconditabacteria*_[SR1]_G_1 (r = 0.628), suggesting niche-specific adaptation in the diabetic microbiome.

#### 3.6.2. H vs. T2DM_P

In the T2DM_P network (Figure 6B), a cohesive and densely connected positive-correlation hub was formed by *Streptococcus*, *Peptostreptococcus*, *Porphyromonas*, *Ottowia*, *Megasphaera*, *Granulicatella*, *Phocaeicola*, *Selenomonas*, *Desulfobulbus*, and *Absconditabacteria*_[SR1]_G_1. In contrast, the Healthy (H) network retained a cooperative commensal hub enriched with *Treponema*, *Filifactor*, *Riemerella*, *Fretibacterium*, *Peptostreptococcaceae_G_6*, *Veillonellaceae_G_1*, *Catonella*, *Neisseria*, *Bacteroidales_G_2*, and *Fusobacterium*, suggestive of a functionally balanced ecological assembly.

Within this dual-hub configuration, *Peptostreptococcus*, *Fretibacterium*, *Filifactor*, and *Treponema* were the dominant keystone species. *Filifactor* genus exhibiting the highest degree centrality and displayed strong and statistically significant positive correlations with *Peptostreptococcaceae_G_4* (*r* = 0.922), *Fretibacterium* (*r* = 0.913), *Catonella* (*r* = 0.852), *Peptostreptococcaceae_G_7* (*r* = 0.806), *Bacteroidales_G_2* (*r* = 0.741), *Peptostreptococcaceae_G_6* (*r* = 0.729), *Porphyromonas* (*r* = 0.639), and *Treponema* (*r* = 0.730). In contrast, *Treponema* exhibited a mixed interactive profile, acting as a secondary connector that engaged in both cooperative and antagonistic interactions. It correlated positively with *Bacteroidales_G_2* (*r* = 0.819, *p* < 0.001), *Fusobacterium* (*r* = 0.704, *p* < 0.01), *Phocaeicola* (*r* = 0.703, *p* < 0.01), *Dialister* (*r* = 0.626, *p* < 0.05), *Catonella* (*r* = 0.684, *p* < 0.01), and *Johnsonella* (*r* = 0.670, *p* < 0.01), while negatively associating with *Granulicatella* (*r* = −0.620, *p* < 0.05). The dual-role genus *Selenomonas* exhibited context-dependent interactions, showing positive correlations with *Stomatobaculum* and *Veillonellaceae*_G_1 (both r = 0.627, r = 0.667) while excluding *Riemerella* (r = −0.606). *Tannerella* acted solely as an antagonist and negatively correlated with *Streptococcus* (r = −0.608) and *Granulicatella* (r = −0.605). In contrast, *Tannerella* acted as a strict antagonist, displaying significant negative correlations with *Streptococcus* (*r* = −0.608) and *Granulicatella* (*r* = −0.605).

Collectively, these networks highlight a shift from a commensal, health-associated interaction web centered on *Treponema*, *Prevotella*, *Catonella*, *Bacteroidales_G_2*, *Filifactor* and *Veillonellaceae* spp. in healthy (H), towards densely connected, pathogen-dominated modules in T2DM and especially T2DM_P, underscoring the pivotal keystone roles of these genera in shaping dysbiotic community structure.

## 4. Discussion

The incidence of Type 2 Diabetes Mellitus (T2DM) varies across different regions due to a multitude of factors, including geographic location, ethnicity, body mass index (BMI), glycemic index, lifestyle, diet, oral health habits, smoking, tobacco chewing, alcohol consumption, and physical exercise [41,42,43]. According to the World Health Organization (WHO), the prevalence of T2DM in low- and middle-income countries has increased at a more rapid pace than in high-income countries [44]. A limited number of studies have explored the oral microbial composition of healthy individuals and those with early-onset T2DM, their comorbidities, periodontitis, and individuals affected by both T2DM and periodontitis [19,20,22]. The present research was conducted as a comprehensive comparative analysis of the oral microbiome in cohorts with T2DM and periodontitis from Mumbai, India. This study provides an integrative perspective on how early-onset T2DM, periodontitis, and their comorbidity-when combined with distinct lifestyle and behavioral profiles are associated with ecological reconfiguration of the gingival plaque microbiome. Age- and sex-matched comparisons across four groups (H, P, T2DM, and T2DM_P), together with detailed characterization of adiposity, systemic features, diet, oral hygiene, and physical activity, enabled robust associations between host- and lifestyle-related risk strata and microbial community composition, diversity, and network architecture. Although α-diversity metrics were largely preserved across groups, β-diversity patterns and co-occurrence network analyses revealed marked disease-associated compositional and interactional shifts, particularly in individuals with combined T2DM and periodontitis (T2DM_P. These findings support an emerging ecological paradigm in which metabolic–periodontal comorbidity is characterized primarily by community restructuring and altered microbial interactions rather than a simple loss of diversity, extending previous observations of dysbiosis in diabetes-associated periodontal disease [45,46,47].

### 4.1. Demographic and Clinical Risk Gradients as the Framework for Microbiome Change

Participants were meticulously matched for age (~35–37 years) and sex distribution, thereby eliminating two primary confounding variables in microbiome composition. Nevertheless, the four groups exhibited significant differences in body mass index (BMI), adiposity patterns, systemic symptoms, and periodontal status. The healthy (H) group was characterized by normal weight, physical activity, absence of cardiometabolic complaints, and intact periodontal health. In contrast, the T2DM and T2DM_P groups demonstrated a substantial prevalence of overweight/obesity and cardiometabolic symptoms, with a short-to-moderate duration of diabetes, aligning with the global trend of increasing early-onset T2DM [7].

Periodontal assessment revealed a distinct gradient of local disease, probing depth, clinical attachment loss, pocket depth, bleeding on probing, and plaque index were markedly elevated in periodontitis (P) and T2DM_P, while remaining low in healthy (H) and T2DM (*p* < 0.0001 for all indices). This resulted in a factorial risk structure, such as healthy (H): metabolically and periodontally healthy reference; periodontitis (P): high periodontal but low metabolic risk; T2DM: high metabolic but low periodontal risk; and T2DM_P: combined high metabolic and high periodontal risk. Such a design is seldom achieved in oral microbiome studies and enabled an examination of how metabolic and local inflammatory risks individually and collectively influence plaque communities, addressing a gap identified in recent systematic reviews on T2DM-periodontitis-microbiota interactions [48,49].

### 4.2. Diet, Lifestyle and Oral Hygiene as Ecological Filters

The groups exhibited significant differences in oral hygiene practices, dietary habits, tobacco and alcohol consumption, and physical activity levels, all of which are recognized as modulators of the oral microbiome [50,51]. Healthy (H) subjects reported engaging in beneficial behaviors, such as brushing their teeth at least twice daily, abstaining from tobacco and alcohol, participating in regular moderate-to-vigorous physical exercise, consuming high amounts of water, and routinely eating fruits, vegetables, and dairy products. These behaviors are consistent with findings from epidemiological and intervention studies, which indicate that a diet rich in plant-based foods, adequate hydration, and low intake of fermentable sugars are associated with a more stable, health-promoting oral microbiota and a reduced risk of periodontal disease [52,53]. Conversely, participants with periodontitis (P) and those with both type 2 diabetes mellitus and periodontitis (T2DM_P) reported brushing only once daily, exhibited heavy plaque accumulation, and had a high prevalence of smoking, smokeless tobacco, and alcohol use, along with frequent consumption of sugar-sweetened beverages and limited intake of fruits and vegetables. Tobacco exposure has been demonstrated to alter the oral microbiome towards a more pro-inflammatory state, characterized by an increased abundance of *Firmicutes* and anaerobic pathogens and a decreased presence of health-associated *Proteobacteria* and commensals [16,54,55,56]. Alcohol consumption and sugary drinks further promote the growth of acidogenic and aciduric taxa, thereby elevating the risk of caries and periodontal disease [57]. In alignment with these observations, our healthy (H) group showed an enrichment of scaffold-forming and early colonizing taxa, such as *Corynebacterium matruchotii*, *Lautropia mirabilis*, and *Capnocytophaga* spp., which are now recognized as key structural components of supragingival plaque and are typically associated with oral health [58]. In contrast, the periodontitis (P) and T2DM_P groups were enriched with classical and emerging periodontopathogens, including *Porphyromonas*, *Tannerella*, *Treponema*, *Fusobacterium*, *Fretibacterium*, and *Peptostreptococcus*, indicative of a proteolytic, inflammation-adapted biofilm characteristic of moderate-to-severe periodontitis [45]. The T2DM group, characterized by substantial metabolic risk but less extreme local behaviors, exhibited an intermediate microbiome with an enrichment of *Porphyromonas pasteri*, *Granulicatella adiacens*, and certain *Streptococcus* lineages, yet lacking the full pathogenic consortium observed in T2DM_P. These patterns are consistent with recent studies indicating that T2DM without periodontitis is associated with subtle but detectable shifts in salivary and subgingival microbiota, which become more pronounced when periodontitis is present [17,19].

### 4.3. Stable α-Diversity with Disease-Specific β-Diversity Restructuring

Across six α-diversity indices (Figure 2A–F) (observed richness, Chao1, Fisher’s α, Shannon, Inverse Simpson, Pielou’s, and Simpson-based evenness), no significant differences were observed between groups (all *p* > 0.05). The within-sample richness and evenness were largely maintained, indicating that the disease states in this cohort involved a rebalancing of taxa rather than a mere loss or gain of diversity. This observation aligns with several recent studies on the oral microbiome in T2DM and periodontitis, which reported modest or inconsistent changes in α-diversity but significant β-diversity and taxonomic differences. In contrast, β-diversity analyses (Figure 3A) using Bray–Curtis distances revealed significant global separation by clinical group, with the most pronounced difference between healthy and T2DM_P groups [45,46,47]. Constrained ordination (CCA) (Figure 3B) demonstrated that metabolic and periodontal factors collectively explained a modest yet statistically significant portion of variance, positioning T2DM samples between healthy (H) and T2DM_P and periodontitis (P) in a partially discrete cluster. These findings are consistent with a recent study indicating that T2DM alters the trajectory of periodontal dysbiosis and that combined disease states occupy distinct compositional niches compared to periodontitis alone [45,46,47]. Collectively, our diversity results support a model in which clinical and behavioral risk factors do not diminish diversity per se but rather drive compositional re-sorting and interactional re-wiring of a relatively stable set of taxa.

### 4.4. Differential Enrichment of Taxa Across Taxonomic Levels Among Study Groups

Phylum-level analysis (Figure 4A) revealed an enrichment of *Firmicutes*, particularly the genera *Streptococcus*, *Veillonella*, *Gemella*, and *Granulicatella*, in both diabetic groups (T2DM and T2DM_P), aligning with findings from previous T2DM studies [20,59]. These genera metabolize simple sugars, producing lactic acid that contributes to enamel erosion and supports aciduric species [60]. Concurrently, a relative depletion of *Bacteroidetes*, *Actinobacteria*, and *Proteobacteria* was observed, which are prevalent in the healthy (H) group [61]; these lineages typically play a role in maintaining biofilm homeostasis through the production of butyrate and short-chain fatty acids (SCFAs). The expansion of *Fusobacteria* in periodontitis (P) is indicative of classic periodontal dysbiosis [17]. The shift in the *Firmicutes*/*Bacteroidetes* ratio has also been associated with systemic metabolic disorders, suggesting a potential oral-gut dysbiosis axis in T2DM [62].

Genus-level analysis revealed distinct mechanisms (Figure 4B). The genera *Streptococcus*, *Leptotrichia*, *Neisseria*, *Veillonella*, *Corynebacterium*, and *Prevotella* have been similarly documented in previous studies [18,21,42]. Genus-level analysis identified distinct group-specific enrichments, healthy (H) plaques were predominantly composed of commensals such as *Corynebacterium*, *Prevotella*, *Capnocytophaga*, *Fusobacteria*, and *Actinomyces*, which likely contribute to homeostatic biofilm functions [63]; the periodontitis (P) group was characterized by pathobionts including *Leptotrichia*, *Neisseria*, *Porphyromonas*, *Tannerella*, and *Peptidiphaga*. The T2DM group was dominated by saccharolytic genera (e.g., *Porphyromonas*, *Gemella*, *Granulicatella*, *Alloprevotella*, and *Abiotrophia*), while the T2DM_P group was characterized by classic periodontal pathogens (e.g., *Streptococcus*, *Veillonella*, *Selenomonas*, *Campylobacter*). These shifts are consistent with observations of altered subgingival and salivary microbiomes in T2DM, characterized by decreased health-associated taxa and increased “red complex” organisms under hyperglycemia [64]. Notably, our findings support the notion that the newly emerging periodontal pathogen *Filifactor* was uniformly detected in both the healthy (H) and periodontitis (P) groups [65,66]. The consistent presence of *Absconditabacteria*_(SR1), *Phocaeicola*, *Acidipropionibacterium*, and *Megasphaera* in both diabetic groups suggests their potential as diabetes-associated biomarkers [64]. Species-level profiling (Figure 5A,B) further refined these distinctions. Healthy (H) plaques were enriched with commensals such as *Corynebacterium matruchotii*, *Lautropia mirabilis*, *Capnocytophaga granulosa*, *Arachnia propionica*, and *Neisseria oralis*, which contribute to network stability through the production of ammonia and H2S, buffering pH, and inhibiting pathogen overgrowth. Periodontitis (P) plaques exhibited blooms of *Selenomonas noxia*, *Leptotrichia shahii*, *Neisseria elongata*, *Veillonella parvula*, and *Capnocytophaga leadbetteri*, key “red complex pathogens” driving pathogenicity via proteolytic enzymes and LPS-mediated host immune response modulation [67]. The T2DM group exhibited elevated levels of *Streptococcus sanguinis*, *Leptotrichia wadei*, *Porphyromonas pasteri*, *Granulicatella adiacens*, and *Gemella morbillorum*, indicating that even in the absence of overt periodontal disease, hyperglycemia fosters acidogenic communities with cariogenic potential [68]. The T2DM_P group was characterized by *Veillonella dispar*, *Streptococcus gordonii*, *Prevotella melaninogenica*, *Kingella oralis*, and *Porphyromonas gingivalis*, suggesting that chronic glycemic dysregulation exacerbates pathogen colonization and tissue invasion [69]. The species *Streptococcus oralis_*subsp.*_dentisani_clade*_058 was uniformly detected in both diabetic groups. Species such as *Scardovia wiggsiae*, *Capnocytophaga gingivalis*, *Bacteroides heparinolyticus*, *Neisseria cinerea*, *Bulleidia extructa*, *Pyramidobacter piscolens*, and *Treponema pectinovorum* were entirely absent from the healthy (H) plaque microbiome.

### 4.5. Keystone Genera and Network Re-Wiring in the Context of Risk

Co-occurrence network analysis (Figure 6A,B), employing stringent Spearman correlation thresholds (|r| ≥ 0.6, FDR-adjusted *p* < 0.05) and focusing on genera present in ≥70% of samples, revealed significant differences in interaction architecture between the groups. This compositionality-aware approach, based on recommendations from network method evaluations, mitigates the risk of spurious correlations and provides more reliable ecological inferences.

In the H vs. T2DM network (Figure 6A), a health-associated hub was identified, comprising *Treponema* spp., *Prevotella*, *Fusobacterium*, *Filifactor*, *Lautropia*, *Veillonellaceae*, and *Fretibacterium*, with *Treponema* and *Veillonellaceae_G_1* exhibiting high degree centrality and serving as connectors among multiple anaerobic genera. The T2DM-biased module, enriched in *Streptococcus*, *Granulicatella*, *Porphyromonas*, *Gemella*, and *Megasphaera*, reflected early metabolic dysbiosis. Positive correlations within these modules suggest cooperative relationships, whereas negative correlations (e.g., between *Lachnoanaerobaculum* and *Streptococcus/Granulicatella*) indicate competitive or exclusionary dynamics that help delineate health- and disease-associated clusters.

In the H vs. T2DM_P network (Figure 6B), the re-wiring was more pronounced. A densely connected pathogenic hub emerged in T2DM_P, centered on *Filifactor*, *Peptostreptococcus*, *Porphyromonas*, *Selenomonas*, *Megasphaera*, *Desulfobulbus*, and *Absconditabacteria (SR1)*, while a residual commensal hub persisted in healthy (H). Within this configuration, *Filifactor* exhibited the highest degree of centrality and strong positive associations with *Porphyromonas*, multiple *Peptostreptococcaceae* sublineages, *Bacteroidales* taxa, and *Treponema*, marking it as a primary keystone. *Treponema* acted as a secondary keystone, linking commensal and pathogenic modules and showing both positive and negative associations (e.g., positive with *Fusobacterium*, *Bacteroidales_G_2*, and *Phocaeicola*; negative with *Granulicatella*). *Tannerella* and *Selenomonas* displayed context-dependent roles, with *Tannerella* being largely antagonistic to *Streptococcus* and *Granulicatella*, and *Selenomonas* cooperating within disease modules and excluding certain health-associated genera. These patterns align with the increasing recognition of *Filifactor*, *Prevotella*, *Treponema*, *Peptostreptococcus*, *Bacteroidales_G*, and *Veillonellaceae_G_1* as key periodontal pathogens and network hubs. These taxa are consistently enriched in deep pockets and severe disease, modulate neutrophil responses, and often co-occur with *Filifactor alocis* and *Porphyromonas gingivalis* (red-complex pathogens) in destructive lesions [70]. Our network findings extend this by demonstrating that *Filifactor*, *Prevotella*, *Treponema*, *Peptostreptococcus*, *Bacteroidales_G*, and *Veillonellaceae_G_1* not only increased in abundance but also became central to the interaction web in T2DM and T2DM_P, coordinating a tightly knit pathogenic consortium under combined metabolic and periodontal stress.

### 4.6. Diet–Microbiome–Host Triad and Implications for Early T2DM with Periodontitis

When contextualized within the broader literature, a coherent narrative emerges. Excessive consumption of dietary sugar and sugar-sweetened beverages is associated with low-grade systemic inflammation, obesity, and Type 2 Diabetes Mellitus (T2DM), and contributes to dysbiosis in both the gut and oral microbiomes. The use of tobacco and alcohol further destabilizes the oral ecosystem, diminishing beneficial taxa and promoting a pro-inflammatory microbiome linked to periodontal degradation [54,56]. Hyperglycemia and insulin resistance exacerbate AGE-RAGE signaling, oxidative stress, and impaired immune resolution, fostering a tissue environment conducive to dysbiotic biofilms dominated by keystone anaerobes, which in turn amplify systemic inflammation. Our cohort exemplifies the interaction of these three components within the T2DM_P group; obesity, high sugar intake, tobacco/alcohol exposure, poor oral hygiene, and established periodontitis co-occur with the most severely altered, pathogen-dominated network and the poorest clinical periodontal scores. In contrast, the healthy (H) group, characterized by a favorable diet, physical activity, and oral hygiene, maintains structural “pillar” taxa such as *Corynebacterium matruchotii*, *Lautropia mirabilis*, *Capnocytophaga granulosa*, *Arachnia propionica*, and *Neisseria oralis*, and exhibits a more balanced network, even in an environment where early-onset T2DM is prevalent.

### 4.7. Clinical and Research Implications

The findings of this study have several practical implications.

Integrated Risk Assessment: The use of simple chairside measures, such as BMI, HbA1c, probing depth, tobacco/alcohol consumption, SSB intake, and brushing frequency, in conjunction with a small panel of microbial markers (e.g., *Filifactor*, *Porphyromonas*, *Treponema*, *Veillonella dispar*, and *C. matruchotii*), could facilitate the stratification of T2DM patients into low, intermediate, and high periodontal risk categories [17].

Targeted Prevention: Interventions that simultaneously address dietary modifications (e.g., reducing sugar-sweetened beverages, increasing plant-based foods), tobacco and alcohol cessation, physical activity, and intensive periodontal care are likely to produce synergistic benefits for both oral and metabolic health. These interventions may also contribute to reversing or stabilizing dysbiosis [56].

Keystone-Focused Therapeutics: Considering the central network role of *Selenomonas*, *Peptostreptococcus*, and associated anaerobes in T2DM_P, strategies that selectively disrupt these keystone taxa or their interactions through antimicrobials, probiotics, prebiotic diets, or biofilm-targeting agents could prove more effective than non-specific microbial suppression.

The strengths of this study include detailed clinical and lifestyle phenotyping, a cross-group design, and the application of conservative, compositionality-aware network analysis. However, the study is limited by its cross-sectional nature, moderate sample size, and reliance on 16S rRNA sequencing, which restricts strain and functional resolution. Future longitudinal and interventional studies employing shotgun metagenomics, metatranscriptomics, and metabolomics, integrated with host immunometabolic profiling, will be crucial to confirm the causal roles of the identified keystone taxa and to evaluate whether modifications in diet, lifestyle, and periodontal status can restore a health-associated plaque ecosystem in individuals with early T2DM.

In conclusion, this study demonstrates that in a relatively young adult cohort, early-onset T2DM and periodontitis do not primarily deplete within-sample diversity. Instead, they reorganize the composition and interaction architecture of plaque communities, particularly under adverse lifestyle and behavioral conditions. The most severe clinical phenotype, T2DM_P, is characterized by dense, keystone-centered pathogenic networks superimposed on a background of metabolic stress and unhealthy diet and lifestyle. This underscores the necessity for truly integrated medical, dental, and lifestyle interventions to mitigate the intertwined burdens of diabetes and periodontal disease.

### 4.8. Contribution to Current Knowledge, Limitations, and Future Perspectives

This study adds to the existing literature by providing one of the few integrative, plaque-based microbiome analyses in early-onset T2DM and T2DM with periodontitis from an Indian urban cohort, explicitly incorporating dietary, lifestyle, metabolic, and periodontal risk gradients within an ecological and network-based framework. Unlike many prior studies that focus primarily on taxonomic shifts or salivary microbiota, this work demonstrates that metabolic–periodontal comorbidity is characterized predominantly by ecological reorganization and network re-wiring rather than α-diversity loss, with the emergence of keystone, inflammation-adapted taxa (e.g., *Filifactor*, *Peptostreptococcus*, *Treponema*, *Veillonellaceae*) under combined metabolic and behavioral stress. These findings refine current models of oral dysbiosis by emphasizing interaction architecture and keystone dynamics as critical features of disease progression.

Several limitations should be acknowledged. The cross-sectional design precludes causal inference, and the moderate sample size, while appropriate for community-level and multivariate analyses, may limit detection of subtle or strain-level effects. Additionally, reliance on 16S rRNA gene sequencing restricts functional and strain-level resolution, and host inflammatory mediators were not directly measured.

Future research should therefore prioritize longitudinal and interventional designs to determine temporal dynamics and causality between metabolic control, lifestyle modification, periodontal therapy, and microbiome restructuring. The integration of shotgun metagenomics, metatranscriptomics, and metabolomics will be essential to elucidate functional pathways and host–microbe interactions underlying keystone behavior. Such studies will be critical for translating microbiome insights into precision risk stratification and targeted, integrated medical–dental–lifestyle interventions for individuals with early-onset T2DM and periodontal disease.

## Figures and Tables

**Table 1:** Periodontal clinical parameters across study groups (Mean + SEM).

Clinical Parameter	Healthy (H) *n* = 10	Periodontitis (P) *n* = 10	Type 2 Diabetes Mellitus (T2DM) *n* = 20	T2DM with Periodontitis (T2DM_P) *n* = 20	*p*-Value
Probing Depth (PD, mm)	2.10 ± 0.12	5.82 ± 0.28	3.21 ± 0.19	6.48 ± 0.31	<0.001
Clinical Attachment Loss (CAL, mm)	0.42 ± 0.08	4.91 ± 0.34	1.98 ± 0.22	5.73 ± 0.37	<0.001
Periodontal Pocket Depth (PPD, mm)	2.02 ± 0.10	6.01 ± 0.30	3.08 ± 0.17	6.62 ± 0.33	<0.001
Bleeding on Probing (BOP, %)	9.4 ± 2.1	68.7 ± 4.9	34.6 ± 3.8	79.2 ± 5.1	<0.001
Plaque Index (PI)	0.62 ± 0.09	2.41 ± 0.16	1.73 ± 0.14	2.68 ± 0.18	<0.001

Statistical analysis: Kruskal–Wallis test followed by Dunn’s post hoc multiple comparison correction. Values are expressed as mean ± SEM.

**Table 2:** Differential mean relative-abundance analysis of Genus among groups (Supplementary Table S4).

Group	Genus (Higher Mean-Relative Abundance Among Groups)
Healthy (H)	*Corynebacterium*, *Prevotella*, *Capnocytophaga*, *Fusobacterium*, *Actinomyces*, *Lautropia*, *Arachnia*, *Rothia*, *Cardiobacterium*, *Filifactor*, *Lachnoanaerobaculum*, *Anaeroglobus*, *Saccharibacteria_(TM7)*_[G-5], *Dialister*, *Ottowia*, *Lachnospiraceae_*[G-3], *Veillonellaceae*_[G-1], *Peptostreptococcaceae_*[G-9], *Riemerella*, *Catonella*, *Peptococcus*, *Weeksellaceae*_[G-1], *Bacteroidetes*_[G-5], *Propionibacteriaceae*_[G-1], *Gracilibacteria*_(GN02)_[G-1], and *Peptostreptococcaceae*_[G-4], *Slackia*, and *Shuttleworthia*.
Periodontitis (P)	*Leptotrichia*, *Neisseria*, *Porphyromonas*, *Saccharibacteria_(TM7)*_[G-1], *Tannerella*, *Peptidiphaga*, *Olsenella*, *Schaalia*, *Peptostreptococcus*, *Bacteroidales*_[G-2], *Aggregatibacter*, *Parvimonas*, *Oribacterium*, *Fretibacterium*, *Filifactor*, *Ruminococcaceae*_[G-1], *Johnsonella*, *Pseudoleptotrichia*, *Peptostreptococcaceae*_[G-7], *Mitsuokella*, *Saccharibacteria*_(TM7)_[G-6], *Peptostreptococcaceae*_[G-6], *Peptoniphilaceae*_[G-1], *Lachnospiraceae*_[G-8], *Bacteroides*, *Peptostreptococcaceae*_[G-1], *Peptostreptococcaceae*_[G-4], *Ruminococcaceae*_[G-2], *Saccharibacteria*_(TM7)_[G-8], *Pseudoramibacter*, *Peptostreptococcaceae*_[G-2], *Pyramidobacter*, *Lachnospiraceae*_[G-7] and *Cryptobacterium*.
Type 2 Diabetes Mellitus (T2DM)	*Porphyromonas*, *Gemella*, *Granulicatella*, *Alloprevotella*, *Abiotrophia*, *Lachnospiraceae*_[G-3], *Eikenella*, *Absconditabacteria*_(SR1)_[G-1], *Phocaeicola*, *Saccharibacteria*_(TM7)_[G-2], *Solobacterium*, *Megasphaera*, *Acidipropionibacterium*, *Butyrivibrio*, *Bacteroidetes*_[G-3], *Mollicutes_*[G-2], and *Bulleidia*.
T2DM associated with P (T2DM_P)	*Streptococcus*, *Veillonella*, *Selenomonas*, *Campylobacter*, *Kingella*, *Lachnospiraceae*_[G-2], *Haemophilus*, *Ligilactobacillus*, *Ruminococcaceae*_[G-1], *Stomatobaculum*, *Scardovia*, *Megasphaera*, *Mitsuokella*, *Absconditabacteria*_(SR1)_[G-1], *Phocaeicola*, *Bifidobacterium*, *Gracilibacteria*_(GN02)_[G-2], *Mycoplasma*, *Lancefieldella*, *Anaerolineae*_[G-1], *Ruminococcaceae*_[G-2], *Acidipropionibacterium*, *Desulfobulbus*, and *Erysipelotrichaceae*_[G-1].

**Table 3:** Differential mean relative-abundance analysis of Species among groups (Supplementary Table S6).

Group	Species (Higher Mean-Relative Abundance Among Groups)
Healthy (H)	*Corynebacterium* spp. *(matruchotii*, *durum)*, *Lautropia mirabilis*, *Capnocytophaga* spp. *(granulosa*, *sputigena)*, *Arachnia* spp. *(propionica*, *rubra)*, *Neisseria oralis*, *Prevotella* spp. *(nigrescens*, *denticola*, *saccharolytica*, *pleuritidis)*, *Capnocytophaga* spp. *(sputigena*, *endodontalis)*, *Rothia* spp. *(dentocariosa*, *aeria)*, *Actinomyces* spp. *(naeslundii*, *massiliensis*, *gerencseriae)*, *Anaeroglobus geminatus*, *Cardiobacterium valvarum*, *Veillonella atypica*, *Dialister invisus*, *Kingella denitrificans*, *Johnsonella ignava*, *Treponema lecithinolyticum*, *Pseudoleptotrichia goodfellowii*, *Slackia exigua*, and *Shuttleworthia satelles*.
Periodontitis (P)	*Leptotrichia shahii*, *Selenomonas noxia*, *Neisseria* spp. *(elongata*, *subflava)*, *Veillonella parvula*, *Capnocytophaga* spp. *(leadbetteri*, *gingivalis)*, *Streptococcus constellatus*, *Cardiobacterium hominis*, *Peptostreptococcus stomatis*, *Fusobacterium nucleatum_*subsp. *(vincentii*, *animalis)*, *Prevotella* spp. *(intermedia*, *micra*, *micans*, *baroniae*, *oralis*, *buccae)*, *Peptidiphaga gingivicola*, *Tannerella forsythia*, *Filifactor alocis*, *Treponema* spp. *(denticola*, *socranskii*, *maltophilum*, *parvum)*, *Bacteroides heparinolyticus*, *Aggregatibacter* sp._HMT_458, *Lachnoanaerobaculum umeaense*, *Fretibacterium fastidiosum*, *Dialister pneumosintes*, *Rothia mucilaginosa*, *Porphyromonas catoniae*, *Olsenella uli*, *Pseudoramibacter alactolyticus*, *Veillonella rogosae*, *Pyramidobacter piscolens*, and *Cryptobacterium curtum*.
Type 2 Diabetes Mellitus (T2DM)	*Streptococcus oralis_*subsp.*_dentisani_clade_058*, *Streptococcus sanguinis*, *Leptotrichia wadei*, *Porphyromonas pasteri*, *Granulicatella adiacens*, *Gemella morbillorum*, *Leptotrichia hongkongensis*, *Streptococcus mutans*, *Alloprevotella tannerae*, *Abiotrophia defective*, *Fusobacterium periodonticum*, *Prevotella* spp. *(oris*, *veroralis*, *nanceiensis)*, *Eikenella corrodens*, *Solobacterium moorei*, *Selenomonas* spp. *(flueggei*, *dianae)*, *Granulicatella elegans*, *Acidipropionibacterium acidifaciens*, *Alloprevotella rava*, *Neisseria cinerea*, *Oribacterium sinus*, and *Bulleidia extructa*.
T2DM associated with P (T2DM_P)	*Veillonella dispar*, *Streptococcus* spp. *(gordonii*, *sobrinus)*, *Selenomonas* spp. *(sputigena*, *artemidis)*, *Prevotella* spp. *(melaninogenica*, *oulorum*, *pallens*, *maculosa*, *salivae*, *dentalis*, *marshii)*, *Kingella oralis*, *Porphyromonas* spp. *(gingivalis*, *endodontalis)*, *Treponema pectinovorum*, *Campylobacter gracilis*, *Haemophilus parainfluenzae*, *Ligilactobacillus salivarius*, *Neisseria bacilliformis*, *Campylobacter concisus*, *Megasphaera micronuciformis*, *Phocaeicola abscessus*, *Bifidobacterium dentium*, *Schaalia cardiffensis*, *Campylobacter curvus*, *Actinomyces* spp. *(oricola*, *dentalis)*, *Mycoplasma salivarium*, *Lancefieldella parvula*, *Oribacterium asaccharolyticum*, *Scardovia wiggsiae*, and *Mycoplasma faucium*.

## Data Availability

All data supporting the findings of this study are available from the corresponding authors upon reasonable request. All raw sequence reads have been deposited in the NCBI Sequence Read Archive (SRA) under BioProject accession number PRJNA1240053 (https://www.ncbi.nlm.nih.gov/sra, accessed on 30 April 2025).

## Supplementary Materials

The following supporting information can be downloaded at: https://www.mdpi.com/article/10.3390/diseases14020038/s1, Supplementary Table S1: Clinical survey metadata; Table S2: Diversity analysis; Table S3: Genus relative abundance; Table S4: Differential mean relative abundance of genus; Table S5: Species relative abundance; Table S6: Differential mean relative abundance of species.
